# Exploring physician agency under demand‐side cost sharing—An experimental approach

**DOI:** 10.1002/hec.4489

**Published:** 2022-04-03

**Authors:** Ge Ge, Geir Godager, Jian Wang

**Affiliations:** ^1^ Department of Health Management and Health Economics University of Oslo Oslo Norway; ^2^ Health Services Research Unit Akershus University Hospital Oslo Norway; ^3^ Dong Fureng Institute of Economic and Social Development, Wuhan University Wuhan China

**Keywords:** demand‐side cost sharing, incentivized laboratory experiment, physician preferences

## Abstract

The assumption of patient‐regarding physicians has been widely adopted in the health economics literature. Physicians' patient‐regarding preferences are often described as the concern for the health benefits of medical treatments, and thus closely related to the norms and ethics of the medical profession. In this paper, we ask whether physicians' patient‐regarding preferences include a concern for their patient's consumption opportunities alongside patient's health benefits. To identify and quantify physicians' preferences, we design and conduct an incentivized laboratory experiment where choices determine separately the health benefits and the consumption opportunities of a real patient admitted to the nearest hospital. We find strong evidence that future physicians care about their patients' consumption opportunities.

## 1 INTRODUCTION

Demand‐side cost sharing in the health sector occurs when a patient is required to pay for a portion of medical treatment costs. Out‐of‐pocket payments by patients can take the form of co‐payments according to a fixed fee schedule or specific co‐insurance rates. Demand‐side cost sharing can be the result of national policy in single‐payer systems such as in the Scandinavian countries, where out‐of pocket payments for various health services are set by the government. In markets where consumers may choose from several health insurance plans, the amount and specific features of demand‐side cost sharing will typically vary substantially between plans, and plans with less cost sharing will necessarily imply higher premiums. Consumers purchasing health insurance in the United States can choose between alternative health insurance plans with different levels of demand‐side cost sharing, and consumers may acquire health insurance with relatively low premiums in exchange for greater cost sharing (Pauly, 2017).

The aim of our study is to contribute to knowledge on physician behavior in the context of demand‐side cost sharing. Under demand‐side cost sharing, the choice of treatment will influence both health benefits and consumption opportunities for patients. The optimal calibration of supply‐ and demand‐side cost sharing has been shown to depend on physician preferences. This paper contributes to the literature on physician preferences in several respects: We acquire data by conducting a laboratory experiment where we attach real monetary incentives to three separate choice attributes. In addition to determining subjects' profit, the choices in the experiment determine the health benefits and the future consumption opportunity of a real patient admitted to the nearest hospital. To the best of our knowledge, this is the first laboratory experiment of its kind. Our experimental design enables the identification and quantification of preferences for the three attributes. Identification of provider preferences are achieved by using a dictator game experiment, which ensures that decision‐makers in our experiment are sovereign in choosing medical treatments.^1^ We strengthen the external validity of our results by recruiting only medical students to participate in the experiment.

Our refutable hypothesis is that medical students ignore the consumption opportunities of the patient when choosing treatment. We find robust evidence suggesting that future physicians' preferences include a concern for patients' consumption opportunities.

The paper proceeds as follows. We relate our study to relevant literature and motivate the research question and experimental methodology in Section 2. In Section 3, we describe the experimental design and protocol. Thereafter, we specify a discrete choice model in Section 4 and report and interpret the results in Section 5. In Section 6, we summarize results and discuss limitations and ideas for further research.

## 2 MOTIVATION AND RELATED LITERATURE

### 2.1 Imperfect agency

Medical treatment choices determine health outcomes and resource use. The economic analysis of health care markets remains a core topic in health economic research. In his seminal paper, Arrow (1963) described the presence of asymmetric information in medical decision‐making as a fundamental aspect of the market for medical care. Physicians are experts holding information superior to patients and insurers, and the physician's relationships with patient and insurer are often characterized by imperfect agency where medical decision‐making are tasks delegated to the physicians. While medical treatment decisions are of great concern to patients and insurers, asymmetric information limits their ability to guide medical decision‐making. As described by Zweifel et al. (2009), patients are often unable to act rationally. In some cases, the illness itself contributes to the limiting of patient sovereignty. Contractibility and information issues limit the payer's opportunities for influencing providers' treatment choices (McGuire, 2000). Imperfect agency is a potential source of market failures with substantial consequences for welfare, and much health economic research has been devoted to studies of remedies for these market failures. Topics in health economics that relate to imperfect agency include *moral hazard* (Pauly, 1968; Zeckhauser, 1970), *adverse selection* (Pauly, 1978), and *supplier induced demand* (Evans, 1974).

Imperfect agency is a challenging element in the joint modeling and analysis of equilibrium in markets for health services and health insurance (Cutler & Zeckhauser, 2000; McGuire, 2012). As noted by Chone and Ma (2011), researchers have not reached a consensus on the formal model of physician agency. However, assuming physicians to be concerned about the well‐being of their patients, and that their medical advice is not guided purely by profit motives has become conventional in mainstream health economics. One may distinguish between two distinctly different approaches to specifying the objective of a patient‐regarding physician: The specification by Farley (1986), which includes *patient utility*, *U*, as an element in the physician's objective, alongside net profits, *π*:

(1) W(π,U),

and the specification by Ellis and McGuire (1990), where patient's health benefit *B*, is included in the physician's objective:

(2) W(π,B).

The specification by Farley (1986) is more general, in that the physician's treatment choice can potentially influence patient utility through differences in out‐of‐pocket payments and differences in health benefits. As noted by Ellis and McGuire (1990), the patient's consumption opportunities are unaffected by medical decisions in the special case where the patient has full insurance. Hence, under full insurance, there is no loss in generality from specifying the physician's objective as in Equation (2). Ellis and McGuire (1990) provide additional motivation for the narrower objective given by Equation (2) noting that medical ethics focus on the patient's health outcomes from treatment rather than patient utility, and that physicians' reputation and risk of malpractice claims also relate to the health outcomes of treatments rather than the overall welfare of the patient. This objective is also supported by Cutler and Zeckhauser (2000), who note that the Hippocratic Oath does not extend to conserving the patient's or society's resources. Modeling provider objective as a combination of profit and health benefit for patients has become the most common approach in the literature. Influential contributions applying this specification include, for example, Ellis and McGuire (1986, 1990); Ma and McGuire (1997); Jack (2005); Léger (2008); Chone and Ma (2011); Chandra et al. (2011); Godager et al. (2015) and Ma and Mak (2019). Theoretical models applying the specification by Ellis and McGuire (1990) have been used to derive the theoretical results that describe advantages of the combined use of demand‐ and supply‐side cost sharing (Cutler & Zeckhauser, 2000; Ellis & McGuire, 1990, 1993). While the specification in Equation (2) is convenient, it remains a restrictive assumption that physicians ignore how treatment choices influence the out‐of‐pocket payments of their patients. The specification by Farley (1986) is more general in that it nests the specification by Ellis and McGuire (1990) as a special case. Experimental data from a carefully‐designed experiment facilitates testing of the hypothesis that physicians ignore patients' consumption opportunities. Further, our model specification in Section 4 enables testing of whether decision‐makers’ are homogeneous in their valuation of patient's consumption opportunities.

### 2.2 Rationale for conducting a choice experiment

We are not the first to study how physicians respond to demand‐side cost sharing. Most studies use field data and the results are mixed. Lundin (2000) found that patients with larger out‐of‐pocket payments are more likely to have generic instead of trade‐name drugs prescribed than those with more costs reimbursed. Hellerstein (1998) did not find strong evidence to suggest that the patient's insurance status systematically influences physicians' prescribing of generic drugs. Hu et al. (2017) found that the introduction of Medicare Part D, which offers more generous coverage for prescription and generic drugs, increased the number of prescription and generic drugs prescribed or continued. The mixed results of physician responses to patients' cost sharing are possibly owning to the challenge of identification. In a field experiment, Lu (2014) addressed a closely related question and found that doctors write significantly more expensive prescriptions to insured patients than to uninsured patients when their profit is linked to patients' drug expenditures. One possible explanation for this result is that doctors may perceive more expensive drugs to be of higher quality. A benefit of conducting experiments in a lab setting is the opportunity of introducing controlled variation in health effects (Hennig‐Schmidt et al., 2011).

If demand responses are imperfect, it is not self‐evident whether observed treatment choices reflect the preference of the patient or preference of the physician. Chandra et al. (2011) describe two ways in which imperfect agency can cause challenges in the interpretation and understanding of observed treatment choices. Preferences of providers and patients are unobserved, and, in addition, it is not clear who has the most influence on the observed outcome. The latter aspect is described in the examples by Ellis and McGuire (1990) and Chandra et al. (2011): A treatment plan that is optimal for the physician might not coincide with the alternative that is optimal for the patient. As illustrated in the example by Chandra et al. (2011, p 405–406), changing financial incentives on supply‐side (provider payment), demand‐side (insurance coverage), or a combination of both, can change whether it is the patient or the doctor who prefers the more costly treatment alternatives. The observed outcomes can be interpreted as the result of bargaining between the parties (Ellis & McGuire, 1990) or the result of a formal or informal partnership (Ma & McGuire, 1997).

Chandra et al. (2011) highlight the need for new theories and empirical research on how treatment decisions are actually made. We believe that the *dictator game* frequently applied in studies of altruism (Almås et al., 2010; Cappelen et al., 2007, 2013; Forsythe et al., 1994) can contribute to such an agenda. These games involve analyzing distributional preferences in a scenario where the decision‐maker is sovereign in the choice of resource allocation. Hence, the use of a dictator games resolves the issue of “who ordered that.”

The study by Hennig‐Schmidt et al. (2011) generalizes the dictator game to a decision situation where the experimental subject's treatment choice simultaneously determines his own profit and the patients' health benefit. Health benefit from medical choices in the experiment by Hennig‐Schmidt et al. (2011) cannot be exchanged for cash directly. Data from experiments using their design has been used to fit empirical specifications of Ellis and McGuire (1990)‐type objective (2) as proposed by Chandra et al. (2011, p. 405). The results by Godager and Wiesen (2013) support the existence of patient‐regarding preferences, and Wang et al. (2020) found that German medical students, Chinese medical students, and Chinese physicians did not differ significantly in their willingness‐to‐pay for patients' health benefit. In this paper, we extend the experimental design of Hennig‐Schmidt et al. (2011) and empirical specification of Chandra et al. (2011) to include the patient consumption opportunities, as proposed by Farley (1986). Similar to Hennig‐Schmidt et al. (2011), we implement a decision context where the physician has all the bargaining power, and the patient is guaranteed to remain passive. In other words, the experimental design and procedure described in the next section provide a situation that is most favorable for physicians to engage in demand inducement (Van De Voorde et al., 2001).

## 3 EXPERIMENTAL DESIGN AND PROCEDURE

### 3.1 General design

Our experimental design is novel in that it combines state‐of‐the‐art methods from the Discrete Choice Experiment (DCE) literature and the experimental health economics literature inspired by Hennig‐Schmidt et al. (2011) who were pioneers in inducing patient‐regarding behavior by means of transfers to a charity caring for real patients. We apply methods from the DCE literature for designing choice menus that are efficient in eliciting preference parameters and the experiment protocol builds on Wang et al. (2020) who were the first to motivate patient‐regarding behavior by treating a real patient at the nearest hospital. Discrete treatment alternatives are described completely in terms of three attributes that are assumed to have impact on physician behavior. In other words, we present treatment alternatives characterized by physician profit, patient health benefit, and patient consumption opportunities. We refrain from introducing non‐incentivized variables, such as “service quantity,” when presenting treatment alternatives to participants. We apply procedures to acquire an efficient design that ensures the identification of preference parameters (Carlsson & Martinsson, 2003; Cheraghi‐Sohi et al., 2008; Huber & Zwerina, 1996). To ensure saliency, all three attributes are incentivized with money. The attributes of health benefit and patient consumption opportunities are incentivized as in Wang et al. (2020) that money representing patient benefit is transferred to the hospital account of a real patient admitted to the nearest hospital. This money can only be used for medical treatment. The transfer of money earmarked for medical treatment is an in‐kind transfer that is clearly distinguishable from a cash transfer.^2^ Money representing patient consumption opportunities in the experiment is given in cash to the same patient. Our design contributes to the small and growing literature on quantifying preference parameters using data from incentivized choice experiments (see, e.g., Ge & Godager, 2021a,2021b; Godager & Wiesen, 2013; Li et al., 2017; Li, 2018; Wang et al., 2020).

In the experiment, each participant plays the role of a physician. Participants make a series of decisions independently and anonymously. Payment to participants depends on their choices in the experiment. We recruit medical students in different semesters to participate in the experiment. A decision task is to choose treatment alternative A or B for a patient who has an endowment of 50 Chinese Yuan (7.55 USD). The patient does not have full insurance and needs to “pay” an out‐of‐pocket fee for the provided treatment. The patient is assumed to be passive and to accept the treatment chosen without interacting with the participant. The choice of treatment A or B simultaneously determines the participant's profit, the patient's health benefit, and the patient's consumption opportunities after the co‐payment. It is public knowledge that demand‐side cost sharing for hospital treatments is common in China.

To be clear, there are no real patients participating in the experiment. To induce patient‐regarding motives, we implemented a procedure similar to that of Wang et al. (2020): The choices made by medical students in the experiment have consequences for one real patient in Qilu Hospital, a hospital located a few minutes' walk from the laboratory.^3^ The patient was chosen randomly from a short list of patients with severe diseases, such as lung cancer, uremia, or other serious illness. The money corresponding to the sum of health benefits provided by all subjects in one of the 23 occasions in the experiment is transferred to the hospital account of this patient, ensuring that the money can only be used for medical treatment. Participants' choices determine the co‐payment and the amount of money available for patient consumption. The latter is transferred in cash directly to the same hospital patient. In the description of the experiment, it is clearly communicated that one unique individual patient would receive all the money.

### 3.2 Choice menus

The choice menus and the specific level of attributes for the alternatives are the result of a Bayesian‐efficient design where so‐called D‐efficiency is optimized. In such designs, the combinations of attribute levels of choice alternatives, pairing of choice alternatives in choice occasions, and the inclusion of choice occasions in blocks are selected in the way that maximizes D‐efficiency. D‐efficient designs excludes scenarios where observed choices contribute with little information about the unknown model parameters. The amount of information one can extract with the given number of choice sets is maximized, and efficient designs facilitate maximal precision in the estimation of the parameters (Moffatt, 2015).

We used the Stata module dcreate (Hole, 2017) to obtain a D‐efficient design.^4^ We employ a block design with four blocks and each block consists of 23 choice menus. Our design was specified to comprise choice menus with two treatment alternatives, and each treatment alternative was characterized by three attributes. We were not aware of any results from experiments of similar efficient designs that could guide us in choosing the number of levels and the range between minimum and maximum levels. We let the maximum level be eight times the minimum level, which is comparable to Hennig‐Schmidt et al. (2011).^5^ Including eight levels for each attribute provides sufficient variations to identify the parameters of a quadratic preference function while maintaining a relatively parsimonious design.

Table 1 shows attributes and their levels. The numerical values of attributes in the choice alternatives presented to participants and reported in Table 1 result from multiplying the levels in our design by 5.^6^ All three attributes are coded as continuous variables. Each attribute has eight levels, ranging from 5 Yuan (0.76 USD) to 40 Yuan (6.04 USD) with a 5‐Yuan (0.76 USD) interval.

**TABLE 1:** Attributes and levels

Attributes	Levels	Coding mode	Expected sign
Your profit	5,10,15,20,25,30,35,40	Continuous	+
Health benefit for the patient	5,10,15,20,25,30,35,40	Continuous	+
Money available to the patient (After co‐payment)	5,10,15,20,25,30,35,40	Continuous	+

Discrete treatment alternatives are characterized by three attributes: “Your profit,” “Health benefit for the patient,” and “Money available to the patient.” “Your profit” indicates how much money a physician would earn from choosing the treatment. “Health benefit for the patient” indicates how much money would be transferred to the patient's hospital account, and “Money available to the patient” indicates how much cash would be transferred directly to the patient when an alternative is chosen. To ensure the clarity and saliency of these attributes, and that the medical students understand the difference between “Health benefit for the patient” and “Money available to the patient,” careful descriptions and test questions were given before the start of the experiment. We explained to medical students that the choice of treatment determines the “Money available to the patient,” which refers to the remaining disposable amount of money that belongs to the patient after paying for the medical treatment. The co‐payment for the treatment can then be calculated by subtracting “Money available to the patient” from the initial endowment of 50 Yuan. By referring to patient's consumption opportunities directly instead of patient's co‐payment, we avoid the discussion of who will receive the co‐payment and the noise from introducing an additional third party in the form of a payer/insurer collecting all or part of the co‐payment.

**FIGURE 1:**
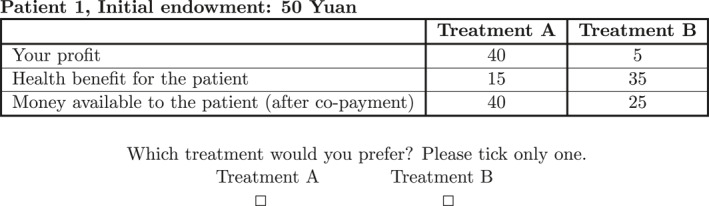
An example of physician's decision task

All choice menus can be grouped by incentive structures. A complete list of all choice menus and a description of incentive categories are presented in Appendix E3. Figure 1 shows an example of a choice menu. This menu represents an incentive structure where both patient and physician are exposed to some degree of cost sharing.^7^ To see this, notice that by choosing Treatment B instead of A, the physician can improve health by sacrificing their own profit. At the same time, Treatment B with larger health effects implies more co‐payment for the patient and less “Money available to the patient (after co‐payment).” In accordance with the convention of avoiding specialized terminologies, such as supply‐side and demand‐side cost sharing in our context, we decided to use neutral layman language that is familiar to medical students to enhance comprehendability (Friedman et al., 1994, p. 52; Jacquemet & l’Haridon, 2018, p. 162).

### 3.3 Experimental protocol

This experiment was conducted at the Lecture Hall of the School of Medicine at Shandong University in China on April 4, 2017. One week before the experiment, 202 medical students were recruited. The Lecture Hall was able to accommodate all participants at the same time. To ensure that there was no interaction between participants, we recruited and trained 10 assistants to supervise the procedure of the experiment.

Upon arrival, the participants were randomly allocated an ID number and led to their seats according to a seating chart. This was to assign participants randomly to blocks, to guarantee that no participant received the same block of choice menus as his or her neighbor to the left or right, and to prevent friends from sitting together. A description of the experiment was then read aloud by the experimenter, and sufficient time was allowed for the participants to ask the assistants for clarifications as well as to ask any questions they had. The participants were then asked three comprehensive questions to familiarize them with the decision tasks. After having made 23 decisions and completing a short questionnaire about their background, each participant received the payment in private. Each participant's payment were comprised of two parts: 25 Yuan (3.77 USD) as compensation for their time spent taking part in the experiment and an amount equals to “Your profit” from a randomly selected decision.^8^ Approximate assessments of the expected duration of the experiment and expected payment to participants were made based on experience. The expected payment to participants was aligned with the amount paid for by a typical student job.

The transfer to the real hospital patient consists of two parts and both are the summed total from all the participants: the money corresponding to the total sum of the “Health benefit for the patient” and the money corresponding to the total sum of the “Money available to the patient.” The amounts were calculated for the randomly drawn choice occasion. The total “Health benefit for the patient” was transferred to the patient's hospital account and could be used only for medical treatment. The total “Money available to the patient” was given as cash to the same hospital patient to use as he or she wished. To validate these two transfers, a monitor was randomly selected from among the experiment's participants (see, Hennig‐Schmidt et al. (2011) who used a monitor in the health economics experiment). The monitor supervised the procedure and executed the transaction together with the experimenter. An additional 30 Yuan (4.53 USD) was paid to the monitor at the end.

The duration of the experiment was 1.5 hours. Participants earned 49.5 Yuan (7.47 USD) on average. In total, 6080 Yuan (917.69 USD) was transferred to the hospital account to pay for medical treatments, and 4635 Yuan (699.59 USD) was given to the patient in cash. Ethical review and approval for the experimental procedure was provided by the Norwegian Social Science Data Services (reference number 53301).

### 3.4 Subject characteristics

To protect the privacy of the participants, we did not collect any identifying information. The description of our study sample in Table 2 is based on the information from the post‐experiment questionnaire. Of the 202 subjects, 72 were males, 129 were females, and one subject did not provide this information. Their ages ranged from 18 to 23 years old, with the majority (67.83%) between 20 and 22 years old. The recruited students were from the study years one to four.^9^ The third and fourth year students accounted for 69.80% of the pool, and they had up to 6 months' experience assisting doctors at the hospitals. The rest of the students were in the first 2 years of study (30.20%) and had voluntary training at the hospital during the summer. Students above year 4 were not available on campus as they were undergoing medical training in hospitals.

**TABLE 2:** Subject characteristics

	Frequency	Percent
Gender		
Male	72	35.64
Female	129	63.86
Unknown	1	0.50
Age		
18	21	10.40
19	31	15.35
20	41	20.30
21	55	27.23
22	41	20.30
23	11	5.45
Unknown	2	0.99
Year of study		
1	46	22.77
2	15	7.43
3	103	50.99
4	38	18.81
Number of individuals = 202		

## 4 A MODEL OF DISCRETE TREATMENT CHOICE

We model a physician's choice of health care treatment alternatives in a situation where the patient does not have full insurance coverage. The choice of medical treatment, therefore, determines the patient's health benefit, *B*, and the patient's out‐of‐pocket payment, *P*, in addition to the physician's net profit, *π*. We specify an objective function, where physicians are assumed to care for the overall well‐being of the patient (Farley, 1986). In practice, our empirical specification extends the specification proposed by Chandra et al. (2011, p. 405), by adding patient consumption opportunities, *C*, to the physician objective. The patient has an endowment equal to *y* ^*o*^. The patient's consumption opportunity is thus equal to the difference between the endowment and the out‐of‐pocket payment: *C* = *y* ^*o*^ − *P*. We start with assuming that the physician's utility specification is linear additive in all choice attributes. Consider a physician choosing one treatment alternative from a choice set with *J* mutually exclusive alternatives. It follows that physician *i*'s utility from providing treatment *j* at choice occasion *t*, denoted as *W*_*ijt*_, can be expressed as:

(3) $$W_{ijt}=\beta_{\pi }\pi_{ijt}+\beta_{B}B_{ijt}+\beta_{C}C_{ijt}+\varepsilon_{ijt},$$

where *i* = 1, 2, …, *I*, *j* = 1, 2, …, *J*, *t* = 1, 2, …, *T*, and *ɛ*_*ijt*_ is a noise term. The noise term is assumed independent and identically type I extreme value distributed which leads to a logit model (McFadden, 1974; Train, 2009). The physician's valuation of profit, patient health benefit, and patient consumption opportunity is captured by the preference parameters *β*_*π*_, *β*_B_ and *β*_C_, respectively.

The functional form of utility functions has been discussed in other economic applications (Keane & Moffitt, 1998; Kim et al., 2016; Koppelman, 1981; Van Soest, 1995), but less attention has been paid to the specifications of utility in the discrete choice literature within the health domain. In health economic applications, the most commonly assumed utility specification is linear additive in all choice attributes as in specification (3).^10^ This type of specification captures only the main effect of each attribute on an individual's decision, which imposes the restriction that the effect of one attribute does not depend on the level of any attribute. In our study, linear utility specification implies that the marginal utility of the physician's profit is constant and does not vary with the level of any one of the three attributes. Despite the challenge that a larger sample is required to estimate a more specific utility function, several studies in the health domain have included attribute‐by‐attribute interactions (Lancsar et al., 2007) and second‐order terms (Kolstad, 2011; Van Der Pol et al., 2010) in the utility specifications and have found significant effects. However, most studies do not discuss the nonlinearities in greater detail. Two recent studies (Holte et al., 2016; van der Pol et al., 2014) investigated the results from different utility specifications, and called for greater attention to questions concerning functional form.

The non‐linear utility specification we choose to use is a quadratic utility with a second‐degree polynomial in all three variables. Suppressing subscripts, it can be written as:

(4) $$W=\beta_{\pi }\pi +\beta_{B}B+\beta_{C}C+\beta_{\pi B}\pi B+\beta_{\text{BC}}BC+\beta_{\pi C}\pi C+\beta_{\pi \pi }\pi^{2}+\beta_{\text{BB}}B^{2}+\beta_{\text{CC}}C^{2}+\varepsilon .$$

According to Taylor's theorem, further expanding the polynomial in the specifications would provide better approximations. Such improvements in approximation of functional forms are costly, however, as more data is required to quantify additional parameters. Furthermore, larger samples and additional parameters also raise computation costs. Hence, a quadratic form is a convenient choice. The linear specification (3) is more restrictive compared to the non‐linear one (4), and we use the former as a baseline for comparison with the other specifications.

We further relax the assumption to allow for heterogeneous preferences among individuals in light of earlier research that found substantial variation in the degree of physicians' other‐regarding preferences (Godager & Wiesen, 2013). Both a discrete representation of unobserved preference heterogeneity (i.e., latent class logit model) and a random parameter logit model estimating parameters of coefficient distributions (i.e., mixed logit model) are applied. While any random utility model can be approximated by a linear (in parameter) mixed logit specification (McFadden & Train, 2000; Train, 2009), choosing the ideal mix and functional form remains a challenge. We estimate both linear and non‐linear functional forms with and without preference heterogeneity. The preferred model is selected based on the Akaike information criterion (AIC) and the Bayesian information criterion (BIC). Our data comprises observed choices of treatment alternatives when available alternatives differ in regard to physician profit, patient benefit, and patient consumption opportunities. Standard conditional logit models, latent class logit model, and mixed logit models are estimated using Stata modules clogit, lclogitml2 (Yoo, 2020), and mixlogit (Hole, 2007).

## 5 RESULTS

### 5.1 Estimation results

We now present the estimation results.^11^ We start with a basic conditional logit model assuming a linear utility specification and homogeneous preferences among individuals (Model 1). We then relax the assumptions of linear utility and homogeneous preferences, respectively. For the former, a conditional logit model with quadratic utility specification is applied (Model 2). For the latter, we used a latent class logit model allowing for various types of preferences among individuals (Model 3). In the end, a flexible mixed logit model accommodating both heterogeneous preferences and nonlinearity in utility is applied (Model 4). For an overview of specifications of all estimated models and corresponding fit criteria, please see Appendix B.

Table 3 presents the estimated results from conditional logit models and a latent class conditional logit model (Models 1, 2, and 3). Both Models 1 and 2 are standard conditional logit models. Model 1 assumes a linear utility in the main effects of the three choice attributes. Estimates from Model 1 indicate that medical students place a positive value on patient consumption opportunities and their own profits when choosing among treatment alternatives, although the patient's health benefit has a much larger impact on choice probabilities. To check whether medical students put the same weight on health benefit and patient consumption opportunities, we conduct a likelihood ratio test.^12^ We reject the hypothesis that participants treat the sum of health benefit and patient consumption opportunities as one single attribute when making treatment decisions (*p* = 0.0000). Model 2 follows a quadratic utility specification allowing for investigation of non‐linearity in variables. In addition to positive marginal utilities, results from Model 2 show that they decrease once we allow for non‐linearity.^13^ Model 3 extends the standard conditional model with linear utility by allowing for a discrete representation of unobserved preference heterogeneity. The optimal number of classes were chosen by examining the AIC and BIC. As a result, the individuals are categorized by four types of preferences. Class 1 consists of only 10.5% of the sample. Individuals in this class put the highest weight on their own profits and the lowest on patient consumption opportunities. Interestingly, patient consumption opportunities does not seem to affect decisions significantly in this class in contrast to the rest of the sample of individuals. In total, 32.3% of the individuals are in Class 2 and they value patient health benefit the most and the patient consumption opportunities the least. Individuals in Class 3 (36.9%) and 4 (20.3%) are similar as both types are most concerned about patient health and least concerned about their own profits. What differentiates them is that the Class 4 type individuals put more than twice as much weight on patient health compared to the Class 3 type individuals. In terms of model fit measured by AIC and BIC, allowing for nonlinear utility improves the goodness of fit, but not as much as the relaxation of homogeneous preference assumption.^14^

**TABLE 3:** Estimation results from conditional logit models and a latent class conditional logit model

Variable	Conditional logit		Latent class conditional logit			
	Model 1	Model 2	Model 3			
			Class1	Class2	Class3	Class4
Physician's profit	0.529***	1.085***	1.596***	0.822***	0.622***	0.534**
	(0.0324)	(0.170)	(0.183)	(0.0649)	(0.0868)	(0.200)
Patient's health benefit	1.401***	2.139***	0.912***	1.697***	2.133***	4.332***
	(0.0640)	(0.243)	(0.143)	(0.126)	(0.158)	(0.886)
Patient's consumption	0.668***	0.761***	0.205	0.486***	1.665***	1.402***
	(0.0509)	(0.217)	(0.117)	(0.0880)	(0.132)	(0.312)
Physician's profit × Patient's health benefit		0.0509**				
		(0.019)				
Patient's health benefit × Patient's consumption		−0.0214				
		(0.021)				
Physician's profit × Patient's consumption		0.0532**				
		(0.017)				
Physician's profit × Physician's profit		−0.140***				
		(0.026)				
Patient's health benefit × Patient's health benefit		−0.140***				
		(0.031)				
Patient's consumption × Patient's consumption		−0.0179				
		(0.028)				
Class share			0.105	0.323	0.369	0.203
Log likelihood	−2097.5	−1985.6	−1714.6			
AIC	4200.9	3989.1	3459.3			
BIC	4222.3	4053.4	3566.3			

*Note*: Standard errors in parentheses and are clustered at the level of the individual. Number of observations: individuals: 202, occasions: 23, decisions: 4645 (One decision was missing from one participating subject).

Abbreviations: AIC, Akaike information criterion; BIC, Bayesian information criterion.

* *p* < 0.05, ** *p* < 0.01, *** *p* < 0.001.

We now relax both assumptions of homogeneous preferences and linear utility by applying a more flexible mixed logit model where we assume random coefficients of variables (Model 4). Table 4 presents estimation results from Model 4. We report the estimated means, standard deviations, and medians (for log‐normal coefficients) of the coefficient distributions. The coefficients *β*_*π*_, *β*_B_, *β*_C_, *β*_*ππ*_, *β*_BB_ and *β*_CC_ are all chosen to be log‐normally distributed because we expect physicians to have positive and decreasing marginal utilities of all variables based on results from the first two models.^15^ The coefficients of *β*_*π*B_, *β*_BC_ and *β*_*π*C_ are assumed to be normally distributed, thereby allowing for the possibility that preferences can be heterogeneous with regard to whether attribute pairs are substitutes or complements. The magnitudes of estimates from our four models are not directly comparable due to different utility specifications and coefficient distributions. Comparing the AIC and BIC in Table 3 with those in Table 4, we see that the goodness of fit improves markedly once we allow for non‐linearity in utility and preference heterogeneity at the same time. Therefore, we focus on estimates from Model 4 when we proceed with post‐estimation results.

**TABLE 4:** Estimation results from a mixed logit model with quadratic utility

Variable	Model 4		
		Estimate	Std. Error†
Physician's profit	Mean	2.079***	(0.337)
	Median	1.880***	(0.366)
	SD	0.980***	(0.130)
Patient's health benefit	Mean	4.045***	(0.415)
	Median	3.973***	(0.424)
	SD	0.775***	(0.198)
Patient's consumption	Mean	1.961***	(0.334)
	Median	1.892***	(0.350)
	SD	0.532***	(0.088)
Physician's profit × Physician's profit	Mean	−0.225***	(0.051)
	Median	−0.222***	(0.050)
	SD	0.040	(0.033)
Patient's health benefit × Patient's health benefit	Mean	−0.252***	(0.058)
	Median	−0.252***	(0.058)
	SD	0.020	(0.041)
Patient's consumption × Patient's consumption	Mean	−0.114*	(0.047)
	Median	−0.085*	(0.042)
	SD	0.099***	(0.024)
Physician's profit × Patient's health benefit	Mean	0.117**	(0.039)
	SD	0.0938*	(0.043)
Patient's health benefit × Patient's consumption	Mean	−0.0244	(0.040)
	SD	0.181***	(0.035)
Physician's profit × Patient's consumption	Mean	0.070**	(0.027)
	SD	0.0331	(0.039)
Log likelihood	−1555.1		
AIC	3146.3		
BIC	3274.7		

*Note*: Coefficients of variables “Physician's profit × Patient's health benefit,” “Patient's health benefit × Patient's consumption,” and “Physician's profit × Patient's consumption” are normally distributed. The remaining coefficients are log‐normally distributed. To facilitate negative second‐order derivatives, the square terms were multiplied by − 1. Model is estimated by means of maximum simulated likelihood, and 3000 Halton draws are used. Number of observations: individuals: 202, occasions: 23, decisions: 4645 (One decision was missing from one participating subject). Standard errors in parentheses and are clustered at the level of the individual.

Abbreviations: AIC, Akaike information criterion; BIC, Bayesian information criterion.

* *p* < 0.05, ** *p* < 0.01, *** *p* < 0.001.

### 5.2 Post‐estimation results

We conduct a post‐estimation analysis on Model 4 which is a quadratic utility specification with random preference coefficients. Suppressing subscript, the first and second order and cross partial derivatives of utility specification Equation (4) are:

(5) $$\begin{array}{ccccccccc} W_{\pi }^{\prime } & = & \beta_{\pi }+\beta_{\pi B}B+\beta_{\pi C}C+2\beta_{\pi \pi }\pi , & W_{\pi \pi }^{\prime \prime } & = & 2\beta_{\pi \pi }, & W_{\pi B}^{\prime \prime } & = & \beta_{\pi B},\\ W_{B}^{\prime } & = & \beta_{B}+\beta_{\pi B}\pi +\beta_{\text{BC}}C+2\beta_{\text{BB}}B, & W_{\text{BB}}^{\prime \prime } & = & 2\beta_{\text{BB}},\; & W_{\text{BC}}^{\prime \prime } & = & \beta_{\text{BC}},\;\\ W_{C}^{\prime } & = & \beta_{C}+\beta_{\text{BC}}B+\beta_{\pi C}\pi +2\beta_{\text{CC}}C,\; & W_{\text{CC}}^{\prime \prime } & = & 2\beta_{\text{CC}},\; & W_{\pi C}^{\prime \prime } & = & \beta_{\pi C}.\; \end{array}$$

The marginal utilities of profit, health benefit, and patient consumption opportunities are denoted as $W_{\pi }^{\prime }$, $W_{B}^{\prime }$, and $W_{C}^{\prime }$. In a non‐linear utility specification, magnitude of marginal utilities depends on both the value of coefficients and the level of variables. The sign of second order derivatives, $W_{\pi \pi }^{\prime \prime }$, $W_{\text{BB}}^{\prime \prime }$, and $W_{\text{CC}}^{\prime \prime }$, indicate whether marginal utilities increase or decrease with the level of variables. The cross partial derivatives, $W_{\pi B}^{\prime \prime }$, $W_{\text{BC}}^{\prime \prime }$, and $W_{\pi C}^{\prime \prime }$, show whether attributes are substitutes or complements.

#### 5.2.1 Simulated marginal utilities

Due to preference heterogeneity, a simulation method is used to illustrate how distributions of marginal utilities vary at different levels of variables. We obtain simulated marginal utility distributions by inserting 100,000 draws from the distributions that are parameterized according to the estimation results in Model 4 into the formulas for the marginal utilities given in Equation (5). We report the simulated median marginal utilities in Table 5.^16^ As expected, the median marginal utilities vary across levels of variables. Table 5, therefore, consists of three panels presenting median marginal utilities at combinations of low (15), middle (20), and high (25) levels of variables. It can be seen that all three median marginal utilities are positive and declining, showing a diminishing marginal utility at the median level.

**TABLE 5:** Simulated median marginal utilities based on estimates from model 4

		*C* = 15			*C* = 20			*C* = 25		
		$W_{\pi }^{\prime }$	$W_{B}^{\prime }$	$W_{C}^{\prime }$	$W_{\pi }^{\prime }$	$W_{B}^{\prime }$	$W_{C}^{\prime }$	$W_{\pi }^{\prime }$	$W_{B}^{\prime }$	$W_{C}^{\prime }$
*π* = 15	*B* = 15	1.50	3.36	1.66	1.53	3.36	1.57	1.57	3.35	1.47
	*B* = 20	1.55	3.11	1.66	1.60	3.11	1.56	1.63	3.10	1.46
	*B* = 25	1.62	2.85	1.65	1.66	2.85	1.55	1.69	2.85	1.46
*π* = 20	*B* = 15	1.28	3.43	1.70	1.31	3.42	1.60	1.35	3.42	1.51
	*B* = 20	1.34	3.18	1.69	1.37	3.17	1.59	1.41	3.16	1.50
	*B* = 25	1.40	2.92	1.68	1.43	2.92	1.58	1.47	2.91	1.48
*π* = 25	*B* = 15	1.05	3.48	1.73	1.09	3.48	1.64	1.12	3.46	1.55
	*B* = 20	1.11	3.24	1.73	1.15	3.23	1.63	1.19	3.22	1.53
	*B* = 25	1.18	2.98	1.72	1.21	2.97	1.62	1.25	2.96	1.52

*Note*: This table presents simulated median marginal utilities $W_{\pi }^{\prime }$, $W_{B}^{\prime }$, and $W_{C}^{\prime }$ at different levels of *π*, B, and C based on estimates from Model 3. 100000 draws were used in the simulation.

#### 5.2.2 Complements or substitutes

Following the definitions by Seidman (1989), we discuss so‐called quantity complements (quantity substitutes) in this paper. For example, *π* and *B* are complements (or substitutes) if an increase in *π* raises (or decreases) the marginal utility of *B*. Hence, two attributes are complements whenever the cross partial derivative in Equation (5) is positive and substitutes if the cross partial derivative is negative.

Since we allow for normal distributed cross partial derivatives, we do not restrict attributes to be either complements or substitutes for all medical students. Two attributes may be complements for some individuals, and substitutes for others. The estimated means of the cross partial derivatives, reported in Table 4, suggest that, *on average*, profit is considered as a complement to both patient benefit and patient consumption opportunities. More specifically, profit is considered as a complement to patient benefit by 89.4% of medical students and to patient consumption by 98.4% of medical students. However, medical students' opinion on the complementarity of health benefit and patient consumption opportunities is more divided, in that 44.6% consider them complements whereas 55.4% consider them substitutes. The average medical students' marginal utility of the patient health benefit is not shown to be significantly affected by the level of patient consumption opportunities.

#### 5.2.3 Simulated marginal rates of substitution

To further study future physicians' trade‐offs between profit, patient health, and patient consumption opportunities, we calculate marginal rates of substitution (MRSs) using the simulated marginal utilities. The individual's MRS for profit and patient health benefit is given by $R_{\pi B}=W_{B}^{\prime }/W_{\pi }^{\prime }$ and expresses how much profit reduction the individual is willing to accept in exchange for an extra unit of patient health benefit, while remaining at the same utility level. Similarly, individual's MRS for profit and patient consumption opportunities is given by $R_{\pi C}=W_{C}^{\prime }/W_{\pi }^{\prime }$, and MRS for patient consumption opportunities and health benefit is written as $R_{CB}=W_{B}^{\prime }/W_{C}^{\prime }$. We report the median of these three MRSs in Table 6, which follows the same format as in Table 5; that is, the MRSs are presented at different combinations of variable level.^17^ The median *R*_*π*C_ take values both larger and smaller than one. This shows that, although they are concerned about patient consumption opportunities after treatment, a median medical student is not in all situations willing to sacrifice more than one unit of profit for one unit of increase in patient consumption opportunities. Additionally, a median medical student would trade off around two units of patient consumption in exchange for one unit of gain in patient health benefit since the median *R*_CB_ is approximately two. Similar to findings in a previous experimental study (Godager & Wiesen, 2013), for a median medical student, approximately two units of profit are willing to be given up for one unit of increase in patient health benefit.^18^

**TABLE 6:** Simulated median marginal rates of substitution based on estimates from model 4

		*C* = 15			*C* = 20			*C* = 25		
		*R*_*π*B_	*R*_*π*C_	*R*_CB_	*R*_*π*B_	*R*_*π*C_	*R*_CB_	*R*_*π*B_	*R*_*π*C_	*R*_CB_
*π* = 15	*B* = 15	2.23	1.08	2.02	2.17	0.99	2.12	2.11	0.89	2.20
	*B* = 20	1.98	1.03	1.88	1.92	0.93	1.96	1.88	0.85	2.03
	*B* = 25	1.74	0.97	1.73	1.70	0.89	1.79	1.65	0.81	1.86
*π* = 20	*B* = 15	2.60	1.26	2.02	2.53	1.15	2.11	2.46	1.05	2.18
	*B* = 20	2.30	1.19	1.88	2.25	1.09	1.96	2.19	1.00	2.03
	*B* = 25	2.03	1.14	1.73	1.98	1.03	1.80	1.92	0.94	1.86
*π* = 25	*B* = 15	2.94	1.42	2.01	2.87	1.30	2.10	2.80	1.20	2.17
	*B* = 20	2.63	1.36	1.87	2.56	1.25	1.95	2.50	1.13	2.02
	*B* = 25	2.31	1.29	1.73	2.26	1.18	1.80	2.20	1.08	1.86

*Note*: This table presents simulated median marginal rates of substitution, *R*_*π*B_, *R*_*π*C_, and *R*_CB_ at different levels of *π*, B, and C based on estimates from Model 3. 100,000 draws were used in the simulation.

The median MRSs depend on variable levels, as one should expect. We find, in general, that median *R*_*π*B_ and median *R*_*π*C_ increase with profit and decrease with patient health and consumption opportunities. The interpretation is that when the profit is relatively high or the patient's utility is low (low health benefits or low consumption opportunities), a median medical student is willing to sacrifice more profit to improve patient utility. The median trade‐off between patient consumption opportunities and benefit, *R*_CB_, does not vary much with changes in profit, but it does increase with patient consumption opportunities and decrease with patient health benefit. Another interesting and intuitive observation is that when profit is high (or low) relative to patient consumption opportunities, a median medical student is willing to give up more (or less) profit than patient consumption for an increase in health benefit.

## 6 DISCUSSION AND CONCLUSION

We ask whether future physicians' patient‐regarding preferences include a concern for patients' consumption opportunities alongside a concern for their health benefit. We conduct a carefully designed choice experiment where three attributes of alternative treatments are incentivized with money. We ensure that participants have a clear understanding of the difference between the variables “Health benefit for the patient” and “Money available to the patient (after co‐payment).” The experimental data enables the identification and quantification of decision‐makers’ valuations of *ceteris‐paribus* changes in the three attributes. The results suggest that medical students are concerned about how their choices of medical treatment affect the consumption opportunities of patients, and this main finding is robust across specifications. The results are intuitive and contribute to the growing experimental evidence on physician preferences.

Achieving saliency in economic experiments is challenging. In our experiment, the three attributes that characterize the choice alternatives are incentivized in terms of cash payments to the participants, cash transfer to a real patient at the nearest hospital, and money deposited to this patient's hospital account for healthcare use. One may argue, however, that a hypothetical layer still remains. Choosing between money transfers for medical treatment cannot be regarded as equivalent to choosing between real medical treatments. In laboratory experiments of the Hennig‐Schmidt et al. (2011) type, money is transferred to a patient‐caring charity to reimburse treatment expenditures (e.g., a cataract treatment). One cannot rule out the possibility that this money transfer is of equivalent value of a direct cash transfer to the patients. The same issue applies for our experiment: Circumstances exist where funds transferred directly to the hospital account might substitute for future deposits that would otherwise have been made by the patient. Importantly, however, our experiment is the first to enable a direct cash transfer (C) in addition to a health benefit transfer (B). Both of these transfers increase the patient's opportunity set. A transfer in cash (C) can, of course, be used to purchase hospital care, or other types of health care services. Since our design includes this opportunity for decision‐makers to affect patient consumption opportunities directly, we can distinguish between patient‐regarding behavior driven by a health benefit motive and patient‐regarding behavior driven by a concern for patient consumption opportunities.

In our experiment, the benefits the patient receives depends on the group's behavior. This arguably differs from a conventional patient‐physician interaction. The consequence might be that the individual participant becomes more, or less, concerned about the patient. The issue remains regardless of whether the beneficiary is a charity or an individual patient. This imperfection can potentially lead to variation in the degree of saliency across the three attributes. As a result, estimated marginal utilities and MRS will be biased. The main result that future physicians are concerned about patient consumption opportunities is robust, regardless of whether there is different degrees of saliency across attributes.

To promote comprehension, we used a neutral layman language when communicating with the participating medical students instead of using specialized economist terminologies, such as *supply‐side cost sharing* or *demand‐side cost sharing*. Our choice of using language that is familiar to participants is in accordance with the conventions in experimental economics (Friedman et al., 1994). Our protocol for describing the experiment to participants was also in accordance with the code of conduct: the experiment was carefully explained to participants, the participants were encouraged to ask clarifying questions, and the participants had to answer comprehension questions correctly before the experiment could be initiated. We have good reasons to believe that the medical students participating in the experiment did have a comprehensive understanding of the choice alternatives in the experiment.

In some circumstances, both doctor and patient can influence choice of treatment. The passive patient in the experiment enables the identification of decision‐makers’ preferences for treatment attributes, as the potential influence of the patient's demand‐response and bargaining power is fixed. While choice scenarios characterized by physician sovereignty do appear in the health sector, and the applicability of the physician sovereignty assumption might extend beyond the treatment of incapacitated patients, one may argue that the imposed physician sovereignty in the dictator game can limit the external validity of our results. For example, the patient has substantial influence on treatment choice in cases of chronic conditions, where the choice of treatment plan will involve physician‐patient interaction to account for the patient's lifestyle choices with regard to, for example, smoking, diet, and exercise. Rather than discussing external validity as a dichotomous aspect that is either present or absent, we discuss when validity is more plausible. One may argue that external validity is less plausible when aiming to apply the results to a broader context (such as “the health care sector”), or to a specific context where patients take an active role in medical decisions. Our experiment does not attempt to provide any new knowledge on physician preference in contexts where the physician‐patient coalition jointly decides on medical treatment. Similarly, one may argue that external validity is more plausible for contexts where it is reasonable to assume physician sovereignty.

We find that medical students act altruistically and care for both the health benefits and consumption opportunities of a patient, even under the most favorable conditions for demand inducement. Future experimental research will contribute to greater knowledge about physicians' preferences in different contexts, and variations in future experimental design will hopefully address limitations in our experiment. Experimental designs that account for variations in patient characteristics, such as type of illness or socioeconomic status, and uncertainty of physicians' knowledge of insurance status are examples of experimental variations that can contribute to new knowledge.

## CONFLICT OF INTEREST

The authors have no conflict of interest. This work has previously appeared in the working papers series of the Department of Health Management and Health Economics, University of Oslo (HERO WP 2019/2) titled “Do physicians care about patients' utility? Evidence from an experimental study of treatment choices under demand‐side cost sharing”.

## Supporting information

Supporting Information S1

Supporting Information S2

## Data Availability

Dataset used in this paper is attached as supplementary material.

## ENDNOTES

^1^
When using the term *sovereignty*, we mean sovereignty in deciding medical treatment. We do not consider the broader kind of sovereignty extending to determining payment schemes and/or the insurance coverage.

^2^
It is a well‐known result from consumer theory that the welfare resulting from a cash transfer of *C* will (weakly) exceed the welfare resulting from an in‐kind transfer with a monetary value of *C*. See, for example, the example provided by Currie and Gahvari (2008). While the welfare effects of a cash transfer weakly dominates the welfare effects of the earmarked transfer, it is another matter whether participants in the experiment actually value the two attributes differently. We return to this question in the results section.

^3^
Hennig‐Schmidt et al. (2011), one of the first health economics experiments incentivizing health benefit, transferred the monetary equivalent of patient health benefit to a German charity. Recent contributions using similar procedure includes for example, Kesternich et al. (2015); Brosig‐Koch et al. (2017a, 2017b); Oxholm et al. (2021, 2019) and Waibel and Wiesen (2021). Wang et al. (2020) modified this procedure so that the money was transferred to the in‐hospital‐account of a cancer patient for his treatment. They argued that the modification was to suit Chinese culture and ensure salience. Firstly, the Chinese population, in general, might have less trust in charitable organizations. Secondly, the medical students are aware that, under the Chinese health insurance plan, a severely ill patient in China most likely bears large out‐of‐pocket expenditures.

^4^
The use of dcreate also ensures that the final experiment design enables identification of desired parameters. In Appendix A, we show that our model parameters are identified with our experiment design.

^5^
In Hennig‐Schmidt et al. (2011), maximum profit is six times minimum profit in, for example, CAP treatment, and patient benefit levels range from zero to 10. While specific field settings exist with either higher or lower ranges, we believe that these ranges have relevance.

^6^
This choice of scaling was to enable us to use real money instead of experimental tokens and to ensure that the expected hourly payment to participants was in line with the payment for a typical student job. Approximate assessments of the expected duration of the experiment were based on experience.

^7^
Supply‐side cost sharing can occur when physicians or hospitals receive a fixed payment, capitation payment, or when marginal cost exceeds fee‐for‐service payment. See, for example, Ellis and McGuire (1990) for the conventional definition of supply‐side and demand‐side cost sharing. By its definition, co‐payment is linearly dependent on the “Money available to the patient (after co‐payment).” The latter is used in describing the experiment to the medical students. Similarly, the reduction in net profit (when providing more patient benefit) is synonymous with supply‐side cost sharing.

^8^
This payment method was chosen to reduce computational costs. An alternative payment approach is to pay for all the decisions made. Charness et al. (2016) survey theoretical predictions and empirical evidence of different payment approaches, and suggest that both methods are effective, in spite of their pros and cons.

^9^
The students invited to participate were undertaking either the 5‐year undergraduate medical degree or the 7‐year program in clinical medicine leading directly to a Master's degree. A modern three‐level medical degree system: Bachelor of Medicine (BM), Master of Medicine (MM), and Doctor of Medicine (DM) was introduced in China in 1981 (Wu et al., 2014). The BM curriculum is the same as the first 5 years of MM at Shandong University.

^10^
In the context of this study, linear or non‐linear utility function means linear or non‐linear in explanatory variables, not parameters. Recently, Andersen et al. (2012) studied several benefits of parametric non‐linear functions.

^11^
In all estimations in this paper, we rescaled all variables by dividing them by 10.

^12^
We test whether Model 1 provides significantly larger explanatory power than a restricted model. Model 1 allows for different coefficients for health benefits and patient consumption opportunities, while the restricted model assumes that the two coefficients are the same, that is, participants put the same weight on health benefits and patient consumption opportunities.

^13^
The magnitudes of marginal utilities depend on the values of all three variables in the non‐linear model. The calculated marginal utilities from Model 2 are positive at all possible combinations of variable levels. The negative coefficients of the second‐order terms indicate decreasing marginal utilities in Model 2.

^14^
We note that the reductions in the information criteria are much larger from homogeneous to heterogeneous preferences than from linear to non‐linear functional form.

^15^
The magnitudes of coefficients of the main effects are much larger than those of the interaction and second order terms; hence, the former almost predominantly decides the sign of the marginal utilities. Therefore, we constrain coefficients of the main effects to be positive.

^16^
The distributions of the estimates used in the simulations are assumed to be independent. A medical student whose marginal utilities take values at or near the median values is therefore a meaningful “type” to describe. A more elaborate overview of marginal utilities at the 25th, 50th, and 75th percentiles of the simulated population is presented in Appendix C.

^17^
A more elaborate overview of marginal rates of substitution at the 25th, 50th, and 75th percentiles of the simulated population is presented in Appendix D. As expected, the simulated median MRSs estimated at *π* = *B* = *C* = 20 are smaller than those from the baseline conditional model with linear utility (Model 1).

^18^
In Godager and Wiesen's (2013) study, the median of estimated *R*_*π*B_ is 1.53 in a mixed logit model and 2.11 in a multinomial logit model.

## APPENDIX A Identification

Over‐parameterization (Biørn, 2016, p107) occurs when the specification includes parameters that cannot be identified, given the variation in the data. In this section, we show how our experimental design enables identification of each of the nine means and nine standard deviations in the specification of the quadratic utility function.

Given the assumption that *β*_*xn*_ is (log) normally distributed with mean *β*_*x*_ and standard deviation $\sigma_{\beta_{x}}$, identification of individual level parameters *β*_*x*1_, *β*_*x*2_, …, *β*_*xN*_ is clearly a sufficient, but not necessary, condition for identification of the parameters of the (log) normal distribution $\left(\beta_{x},\sigma_{\beta_{x}}\right)$. Hence, if the design enables identification of nine parameters per decision‐maker, the design will certainly enable identification of the means and standard deviations of nine random coefficients of our mixed logit model specification. We now deduct the so‐called rank condition for identification of the individual‐level‐parameters in Equation (4).

Assume a linear in parameters utility function with one parameter. The deterministic utility of individual *n* (*n* = 1, 2…, *N*) when selecting alternative *j* (*j* = *A*, *B*) in occasion *t* (*t* = 1, 2, …, *T*) is:

(6)
$$W_{njt}=\beta_{xn}X_{jt}$$

When the probability that individual *n* chooses *A* rather than *B* is given by the logit formula, the odds in occasion *t* becomes:

(7)
$$\frac{P_{nAt}}{P_{nBt}}=e^{\beta_{xn}\left[X_{At}-X_{Bt}\right]},$$

and the log of the odds becomes:

(8)
$$\ln\left(P_{nAt}/\left(1-P_{nAt}\right)\right)=\beta_{xn}\Delta X_{t},$$

where we introduce the definition Δ*X*_*t*_ ≡ *X*_*At*_ − *X*_*Bt*_. With three attributes, the quadratic utility function comprises nine parameters. Let Δ ***X*** denote the 9 × 9 matrix of attribute differences for the three attributes, the corresponding three square terms, and their three interaction terms in Equation (4) for *t* = 1, 2…9, that is, for nine specific choices occasions. ***β*** denotes the 1 × 9 vector of unknown parameters in Equation (4), and **b** denotes the 9 × 1 vector comprising the log of odds for the nine choice occasions. We may now generalize and write Equation (8) as a system of equations:

(9)
$$b=\Delta X\beta^{\prime }$$

The equation system (9) includes nine equations, one for each of nine occasions. Let Δ**Xb** denote the so‐called *augmented matrix* of the system (9) which include Δ ***X*** as the first nine columns and **b** as the tenth column (Sydsæter et al., 2005). The rank condition states that a necessary and sufficient condition for (9) to be consistent (have at least one solution, ***β*** *) is that the two matrices Δ**Xb** and Δ ***X*** have the same rank (Sydsæter et al., 2005, p21). Given that there is variation in the elements of **b**, it suffices to check if Δ ***X*** is non‐singular. If Δ ***X*** is found to be non‐singular, the two matrices Δ**Xb** and Δ ***X*** must have the same rank. We see that the question of identification rests on whether the experiment design provides (at least) nine occasions where the attribute difference matrix, Δ ***X***, is non‐singular. In other words, a sufficient condition for identification of all nine parameters is that there exists nine choice occasions in the experiment where there is no linear dependence between any of the nine differences.

We have checked the rank condition for identification of the nine individual level parameters of the quadratic utility function.^19^ The experimental design ensures identification of all parameters to be estimated: The symmetric 9 × 9 difference‐matrix Δ ***X*** formed by the *first* nine tasks in block *i* is non‐singular for *i* = 1, 2, 3, 4. Hence, the nine parameters of the quadratic utility function is identified at the level of the individual decision‐maker, regardless of which of the four blocks the individual is assigned to. Since the difference‐matrix formed by the *last* nine occasions in block *i* is also non‐singular for *i* = 1, 2, 3, 4, we know that the nine parameters of the quadratic utility function of decision‐maker *n* is *over‐identified* (Cameron & Trivedi, 2005, p35), regardless of which of the four blocks the individual is assigned to. This over‐identification result implies that the experimental design enables identification of individual level parameters *and* their corresponding standard errors.

We do not aim to estimate individual‐level utility functions in this paper. Our objective is to obtain estimates of the parameters of the (log) normal distribution of the nine random coefficients. Since identification follows by implication from over‐identification of individual level parameters and the parametric assumptions of our mixed logit model, our careful check to confirm absence of collinearity provides documentation that the design enables identification of the distribution parameters of the random coefficients in Equation (4). This result should not come as a surprise, since the procedure we applied for selecting D‐efficient design prevents variables from becoming collinear or strongly correlated in the empirical specification.

## APPENDIX B Model specifications

Table B1 gives a summary of all the estimated models with log likelihood, AIC and BIC listed at the end. Panel A and B respectively presented results from eight linear and five non‐linear utility specifications. The selected models discussed and compared in the article are model A, B, I and M, of which M is the preferred model.

**TABLE B1:** Log‐likelihood and information criteria from all model specifications

Panel A: Linear utility *U* = *β*_*π*_ *π* + *β*_B_ *B* + *β*_C_ *C* + *ɛ*			
Specification of heterogeneity, if present	**Log likelihood**	**AIC**	**BIC**
(A) No heterogeneity **[Model 1]**	−2097.5	4200.9	4222.3
(B) Discrete preference heterogeneity **[Model 3]**	−1714.6	3459.3	3566.3
(C) *β*_*B*_, *β*_*C*_ (Normal)	−1801.1	3612.1	3647.8
(D) *β*_*π*_, *β*_*B*_, *β*_*C*_ (Normal)	−1742.9	3497.7	3540.5
(E) *β*_*π*_, *β*_*B*_, *β*_*C*_ (Correlated, normal)	−1718.8	3455.7	3519.9
(F) *β*_*B*_, *β*_*C*_ (Log‐normal)	−1816.7	3643.5	3679.1
(G) *β*_*π*_, *β*_*B*_, *β*_*C*_ (Log‐normal)	−1730.6	3473.2	3516.0
(H) *β*_*π*_, *β*_*B*_, *β*_*C*_ (Correlated, log‐normal)	−1713.7	3445.5	3509.7

**Panel B: Quadratic utility**			
*U* = *β*_*π*_ *π* + *β*_B_ *B* + *β*_C_ *C* + *β*_*π*B_ *πB* + *β*_BC_ *BC* + *β*_*π*C_ *πC* + *β*_*ππ*_ *π* ^2^ + *β*_BB_ *B* ^2^ + *β*_CC_ *C* ^2^ + *ɛ*			
**Specification of heterogeneity, if present**	**Log likelihood**	**AIC**	**BIC**
(i) No heterogeneity **[Model 2]**	−1985.6	3989.1	4053.4
(j) *β*_*π*_, *β*_*B*_, *β*_*C*_ (Normal)	−1575.2	3174.4	3260.0
(k) *β*_*π*_, *β*_*B*_, *β*_*C*_ (Log‐normal)	−1565.4	3154.8	3240.5
(l) All coefficients (normal)	−1561.2	3158.4	3286.9
(m) *β*_*π*B_, *β*_BC_, *β*_*π*C_, (Normal), *β*_*π*_, *β*_B_, *β*_C_, *β*_*ππ*_, *β*_BB_, *β*_CC_ (log‐normal) **[Model 4]**	−1555.1	3146.3	3274.7

*Note*: Individual, alternative, and time subscripts are suppressed in the utility functions.

## APPENDIX C Simulated marginal utilities

Table C1 is an overview of marginal utilities at different levels of *π*, B, and C for the 25th, 50th and 75th percentile of the simulated population. The simulation is based on estimates from Model 4 and 100,000 draws are used in the simulation process.

**TABLE C1:** Simulated marginal utilities based on estimates from Model 4

*π* = 15		*C* = 15			*C* = 20			*C* = 25		
		$W_{\pi }^{\prime }$	$W_{B}^{\prime }$	$W_{C}^{\prime }$	$W_{\pi }^{\prime }$	$W_{B}^{\prime }$	$W_{C}^{\prime }$	$W_{\pi }^{\prime }$	$W_{B}^{\prime }$	$W_{C}^{\prime }$
*B* = 15	25%	0.99	2.84	1.25	1.02	2.81	1.14	1.06	2.77	1.02
	50%	1.50	3.36	1.66	1.53	3.36	1.57	1.57	3.35	1.47
	75%	2.17	3.94	2.10	2.21	3.96	2.01	2.24	3.98	1.93
*B* = 20	25%	1.04	2.59	1.22	1.08	2.55	1.09	1.11	2.52	0.97
	50%	1.55	3.11	1.66	1.60	3.11	1.56	1.63	3.10	1.46
	75%	2.23	3.70	2.12	2.27	3.71	2.03	2.30	3.73	1.96
*B* = 25	25%	1.10	2.33	1.16	1.14	2.30	1.05	1.17	2.26	0.93
	50%	1.62	2.85	1.65	1.66	2.85	1.55	1.69	2.85	1.46
	75%	2.31	3.44	2.15	2.34	3.46	2.06	2.38	3.48	1.98

*π* = 20		*C* = 15			*C* = 20			*C* = 25		
		$W_{\pi }^{\prime }$	$W_{B}^{\prime }$	$W_{C}^{\prime }$	$W_{\pi }^{\prime }$	$W_{B}^{\prime }$	$W_{C}^{\prime }$	$W_{\pi }^{\prime }$	$W_{B}^{\prime }$	$W_{C}^{\prime }$
*B* = 15	25%	0.77	2.90	1.29	0.80	2.87	1.17	0.83	2.83	1.06
	50%	1.28	3.43	1.70	1.31	3.42	1.60	1.35	3.42	1.51
	75%	1.94	4.01	2.13	1.99	4.03	2.05	2.02	4.05	1.98
*B* = 20	25%	0.82	2.64	1.25	0.85	2.61	1.13	0.89	2.57	1.01
	50%	1.34	3.18	1.69	1.37	3.17	1.59	1.41	3.16	1.50
	75%	2.01	3.76	2.16	2.05	3.77	2.07	2.09	3.79	1.99
*B* = 25	25%	0.87	2.39	1.20	0.91	2.36	1.08	0.94	2.32	0.96
	50%	1.40	2.92	1.68	1.43	2.92	1.58	1.47	2.91	1.48
	75%	2.08	3.51	2.18	2.11	3.53	2.09	2.16	3.54	2.01

*π* = 25		*C* = 15			*C* = 20			*C* = 25		
		$W_{\pi }^{\prime }$	$W_{B}^{\prime }$	$W_{C}^{\prime }$	$W_{\pi }^{\prime }$	$W_{B}^{\prime }$	$W_{C}^{\prime }$	$W_{\pi }^{\prime }$	$W_{B}^{\prime }$	$W_{C}^{\prime }$
*B* = 15	25%	0.54	2.94	1.32	0.57	2.92	1.21	0.60	2.87	1.09
	50%	1.05	3.48	1.73	1.09	3.48	1.64	1.12	3.46	1.55
	75%	1.73	4.07	2.17	1.77	4.09	2.09	1.81	4.10	2.01
*B* = 20	25%	0.59	2.70	1.28	0.63	2.66	1.17	0.66	2.62	1.04
	50%	1.11	3.24	1.73	1.15	3.23	1.63	1.19	3.22	1.53
	75%	1.79	3.83	2.19	1.84	3.84	2.11	1.87	3.86	2.03
*B* = 25	25%	0.65	2.44	1.23	0.68	2.40	1.11	0.72	2.36	1.00
	50%	1.18	2.98	1.72	1.21	2.97	1.62	1.25	2.96	1.52
	75%	1.87	3.57	2.22	1.90	3.59	2.13	1.94	3.60	2.05

*Note*: This table presents marginal utilities $W_{\pi }^{\prime }$, $W_{B}^{\prime }$, and $W_{C}^{\prime }$ at different levels of *π*, B, and C for the 25th, 50th, 75th percentiles of the preference distributions.

## APPENDIX D Simulated marginal rates of substitution

Table D1 is an overview of MRS at different levels of *π*, B, and C for the 25th, 50th and 75th percentile of the simulated population. The simulation is based on estimates from Model 4 and 100,000 draws are used in the simulation process.

**TABLE D1:** Simulated marginal rates of substitution based on estimates from Model 4

*π* = 15		*C* = 15			*C* = 20			*C* = 25		
		*R*_*π*B_	*R*_*π*C_	*R*_CB_	*R*_*π*B_	*R*_*π*C_	*R*_CB_	*R*_*π*B_	*R*_*π*C_	*R*_CB_
*B* = 15	25%	1.48	0.67	1.51	1.44	0.59	1.54	1.40	0.52	1.55
	50%	2.23	1.08	2.02	2.17	0.99	2.12	2.11	0.89	2.20
	75%	3.48	1.75	2.79	3.37	1.61	3.02	3.25	1.47	3.23
*B* = 20	25%	1.32	0.63	1.37	1.28	0.56	1.39	1.25	0.49	1.40
	50%	1.98	1.03	1.88	1.92	0.93	1.96	1.88	0.85	2.03
	75%	3.08	1.66	2.67	2.96	1.52	2.88	2.88	1.41	3.08
*B* = 25	25%	1.15	0.59	1.22	1.13	0.52	1.23	1.09	0.45	1.24
	50%	1.74	0.97	1.73	1.70	0.89	1.79	1.65	0.81	1.86
	75%	2.69	1.59	2.53	2.60	1.47	2.72	2.54	1.36	2.92

*π* = 20		*C* = 15			*C* = 20			*C* = 25		
		*R*_*π*B_	*R*_*π*C_	*R*_CB_	*R*_*π*B_	*R*_*π*C_	*R*_CB_	*R*_*π*B_	*R*_*π*C_	*R*_CB_
*B* = 15	25%	1.64	0.74	1.51	1.60	0.67	1.54	1.56	0.59	1.55
	50%	2.60	1.26	2.02	2.53	1.15	2.11	2.46	1.05	2.18
	75%	4.38	2.19	2.77	4.25	2.01	2.99	4.10	1.84	3.20
*B* = 20	25%	1.46	0.70	1.37	1.43	0.62	1.40	1.39	0.55	1.40
	50%	2.30	1.19	1.88	2.25	1.09	1.96	2.19	1.00	2.03
	75%	3.86	2.08	2.65	3.70	1.90	2.85	3.58	1.74	3.06
*B* = 25	25%	1.28	0.66	1.23	1.26	0.58	1.25	1.22	0.51	1.25
	50%	2.03	1.14	1.73	1.98	1.03	1.80	1.92	0.94	1.86
	75%	3.35	1.96	2.52	3.23	1.79	2.71	3.15	1.66	2.91

*π* = 25		*C* = 15			*C* = 20			*C* = 25		
		*R*_*π*B_	*R*_*π*C_	*R*_CB_	*R*_*π*B_	*R*_*π*C_	*R*_CB_	*R*_*π*B_	*R*_*π*C_	*R*_CB_
*B* = 15	25%	1.70	0.77	1.51	1.68	0.70	1.54	1.66	0.62	1.56
	50%	2.94	1.42	2.01	2.87	1.30	2.10	2.80	1.20	2.17
	75%	5.41	2.72	2.73	5.24	2.48	2.95	5.05	2.28	3.17
*B* = 20	25%	1.53	0.74	1.37	1.52	0.67	1.40	1.49	0.59	1.40
	50%	2.63	1.36	1.87	2.56	1.25	1.95	2.50	1.13	2.02
	75%	4.77	2.56	2.62	4.59	2.34	2.82	4.43	2.14	3.01
*B* = 25	25%	1.37	0.70	1.24	1.35	0.62	1.25	1.32	0.55	1.26
	50%	2.31	1.29	1.73	2.26	1.18	1.80	2.20	1.08	1.86
	75%	4.11	2.39	2.50	4.01	2.21	2.70	3.86	2.02	2.89

*Note*: This table presents marginal rates of substitution, *R*_*π*B_, *R*_*π*C_, and *R*_CB_ at different levels of *π*, B, and C for the 25th, 50th, 75th percentiles of the preference distributions.

## APPENDIX E Experimental design

### E.1 Description of the experiment

**General information**

Welcome to our experiment. This experiment is part of a research collaboration between the Universities of Shandong (China), Oslo (Norway), and Cologne (Germany).

In the following experiment, you will make several decisions. Following the instructions and depending on your decisions, you can earn money. It is therefore very important to read the description carefully.

Your decisions are anonymous and will be kept strictly confidential. During the experiment you are not allowed to talk to any other participant. Whenever you have a question, please raise your hand. The experimenter will answer your question in private. If you disregard these rules you can be excluded from the experiment without receiving any payment.

All amounts in the experiment are stated in Chinese Yuan (RMB). At the end of the experiment, you will be paid in cash.

After the experiment, we will kindly ask you to complete a short questionnaire and you will get 25 Yuan for carefully completing the experiment and questionnaire.

The experiment will take approximately 1 hour and a half.

**Decision situations in the experiment**

During the entire experiment you are in the role of a physician. You decide on the treatment options – Treatment A or Treatment B – of 23 abstract patients. There are no real patients participating in this experiment, but a real patient outside the experiment will be affected by your decisions.

The Treatment A and B differ in terms of **Your profit**, **Health benefit for the patient** and **Money available to the patient (after co‐payment)**. We now explain the three elements one by one:

**Your profit** indicates how much money you would earn from choosing the treatment.

**Health benefit for the patient** is the patient's expected gain in health status, measured in money, from your choice of treatment.

Each patient you are treating has the same amount of money initially: 50 Yuan. The patients are **not** fully insured. This means that they have to pay a certain amount of co‐payment for the treatment. **Money available to the patient (after co‐payment)** therefore indicates the amount of money that remains with patient, after the co‐payment. The patient can spend the remaining amount of money on any feasible consumption. Hence, your decision on the treatment not only determines your own profit, but also the patient's health benefit and consumption level after co‐payment. Note that, in this experiment, we do not consider third party insurer's payment for the treatment.

Consider the following example:

Patient 6

With an initial endowment of 50 Yuan

**Table:**

	Treatment A	Treatment B
Your profit	15	40
Health benefit for the patient	25	5
Money available to the patient (after co‐payment)	10	30

Which treatment would you prefer, Treatment A or Treatment B? Please tick only one.

This patient has an initial money endowment of 50 Yuan. You are asked to choose either Treatment A or Treatment B. If you choose Treatment A, you will get 15 Yuan profit. If you choose Treatment B, you will get 40 Yuan profit which is 25 Yuan more than in Treatment A. For the patient, Treatment A gives a health benefit valued at 25 Yuan, and this is 20 Yuan more than in Treatment B. At the same time, the patient has to pay 40 Yuan co‐payment for Treatment A and 20 Yuan for Treatment B. Equivalently, the money available to the patient after co‐payment is 10 Yuan if Treatment A is chosen, and the money available to the patient after co‐payment is 30 Yuan if Treatment B is chosen. You can calculate the co‐payment by subtracting the Money available to the patient (after co‐payment) from the initial endowment of 50 Yuan.

Once you make your decision, tick the box under your preferred treatment.

**The payments in the experiment**

After everyone have completed the booklet with decision tasks and questionnaire, an assistant will collect the booklet. After collecting all of the booklets, one out of your 23 decisions will be drawn randomly. The payoff for you and the patient will be based on this randomly drawn decision.

There are no real patients participating in this experiment, but your decision on the abstract patient will benefit a real patient in Qilu Hospital. This real patient is randomly chosen from a list of admitted patients who have serious diseases (e.g., lung cancer, uremia, colon cancer or other serious illness) and have to bear a co‐payment for his or her medical treatment.

**The payment you receive**: The amount of **Your profit** from the randomly drawn decision and the participation fee, will be given to you in cash at the end of the experiment.

**The transfers to the patient**: This transfer consists of two parts. The amount of **Health benefit for the patient** from the decision will be transferred to the patient's hospital account. It can only be used for medical treatment for the patient. At the same time, the amount of **Money available to the patient (after co‐payment)** will be given to the patient as cash at his or her disposal.

**Procedural details**

After the experiment, one of you will be randomly chosen as a monitor who will supervise the transactions to the patient. The monitor and the experimenter will both go to Qilu hospital and supervise the process of transferring the money to the patient's hospital account and give the cash directly to the patient. The visit to Qilu hospital will take place after the experiment. The monitor will receive an hourly payment of 30 Yuan in addition to the payment from the experiment. The monitor verifies, by a signed statement, that the procedure described above is carried out.

After the experiment, the hospital will indicate in an anonymous way to the researchers which medical treatments have been conducted for the randomly chosen patient using the transferred money to the patient's hospital account. This document will made accessible to participants of this experiment upon request.

Now, please answer some questions familiarizing you with the decision situation. The experiment will only start when all subjects have answered the comprehension questions correctly. After your 23 decisions, please answer a short questionnaire about your background.

### E.2 Comprehension questions

Now, please answer the following three questions to familiarize yourself with the decision situation. Once you are done, please raise your hand, and one of our experimenters will check your answers.

1. Are the following statements correct or incorrect?
  A: All 23 decisions are equally important, because one randomly drawn decision will determine my payment.
    □ Correct □ Incorrect
  B: My decision on the treatment will benefit a real patient.
    □ Correct □ Incorrect
  C: The patients are fully insured, so they don't bear any co‐payment for the treatment.
    □ Correct □ Incorrect
2. Consider the following choice situation. Patient 1 with an initial endowment of 50 Yuan.
  **Table:**
  	Treatment A	Treatment B
  Your profit	10	20
  Health benefit for the patient	30	25
  Money available to the patient (after co‐payment)	15	15
  Which treatment would you prefer, Treatment A or Treatment B? Please tick only one.
  Please fill in the blanks with correct numbers.
  If you choose Treatment A, you will get ______Yuan profit, the patient will gain ______Yuan in health benefit and he or she has to pay ______Yuan co‐payment out of pocket, leaving him or her with a remaining amount of ______Yuan.
  If you choose Treatment B, you will get ______Yuan profit, the patient will gain ______Yuan in health benefit and he or she has to pay ______Yuan co‐payment out of pocket, leaving him or her with a remaining amount of ______Yuan.
3. Consider another choice situation. Patient 2 With an initial endowment of 50 Yuan
  **Table:**
  	Treatment A	Treatment B
  Your profit	20	35
  Health benefit for the patient	30	20
  Money available to the patient (after co‐payment)	15	10
  Which treatment would you prefer, Treatment A or Treatment B? Please tick only one.
  Please fill in the blanks with correct numbers.
  If you choose Treatment A, you will get ______Yuan profit, the patient will gain ______Yuan in health benefit and he or she has to pay ______Yuan co‐payment out of pocket, leaving him or her with a remaining amount of ______Yuan.
  If you choose Treatment B, you will get ______Yuan profit, the patient will gain ______Yuan in health benefit and he or she has to pay ______Yuan co‐payment out of pocket, leaving him or her with a remaining amount of ______Yuan.

This is the end of the comprehension questions. Please raise your hand and wait for an experiment assistant to check your answers.

### E.3 Choice menus and incentive categories

We employed a block design with four blocks. Each block includes 23 choice menus. In each block, 18 choice menus are unique to the block whereas five are overlapping across blocks. There are 77 unique choice menus overall. Table E1 below presents all the choice menus in the experiment.

**TABLE E1:** Choice menus

Choice Menu	Treatment alternative	Block 1			Block 2			Block 3			Block 4		
		*π*	*B*	*C*	*π*	*B*	*C*	*π*	*B*	*C*	*Π*	*B*	*C*
1	A	40	15	35	10	40	5	15	40	5	40	10	20
	B	10	20	30	15	20	15	10	5	10	20	40	5
2	A	35	5	15	10	40	5	5	5	40	40	30	5
	B	15	5	40	30	15	5	10	10	25	5	15	15
3	A	40	5	5	35	15	40	20	40	40	10	40	10
	B	15	10	15	5	30	35	25	5	40	15	10	15
4	A	40	35	20	25	10	5	40	5	5	5	5	40
	B	35	10	40	40	5	5	20	15	5	5	20	15
5	A	5	25	10	5	5	10	25	20	30	15	10	25
	B	5	5	40	30	5	5	40	30	5	40	10	5
6	A	40	5	40	20	20	10	25	10	10	40	15	40
	B	40	30	15	5	40	5	40	5	5	5	35	25
7	A	5	40	5	5	35	40	40	40	15	5	10	40
	B	5	20	20	35	30	5	30	15	25	5	40	5
8	A	10	5	40	40	5	5	40	5	5	10	10	25
	B	20	10	20	10	20	5	15	5	20	5	5	40
9	A	5	10	40	35	5	35	20	40	10	5	40	40
	B	10	20	25	15	40	30	35	5	40	25	20	25
10	A	5	15	40	40	25	5	15	30	35	40	40	25
	B	5	40	10	5	15	10	40	10	40	20	30	40
11	A	40	5	5	15	5	35	30	10	40	40	5	5
	B	20	10	10	40	5	15	5	25	40	20	15	10
12	A	40	10	5	30	5	40	40	25	40	5	40	5
	B	25	15	10	20	40	40	10	35	25	15	20	10
13	A	35	40	10	20	10	15	5	40	40	15	5	15
	B	20	20	40	5	5	40	35	10	15	5	5	35
14	A	30	35	40	15	40	5	40	5	5	10	40	5
	B	35	30	5	10	5	40	5	5	35	35	15	5
15	A	35	40	40	5	40	40	10	5	40	35	5	30
	B	25	35	15	40	20	25	25	15	20	30	40	10
16	A	10	40	5	40	5	40	5	40	5	40	35	5
	B	35	5	5	20	40	20	10	25	10	40	20	35
17	A	30	40	5	5	40	15	40	40	25	40	5	40
	B	20	30	35	5	10	35	25	25	30	35	30	10
18	A	40	40	5	30	5	40	5	10	40	40	25	5
	B	10	20	30	20	20	5	5	40	10	5	35	40
19	A	10	15	15	5	40	5	5	5	30	35	5	40
	B	5	40	5	15	15	15	40	5	5	30	30	40
20	A	20	15	5	35	40	40	35	40	40	35	40	40
	B	40	5	5	25	35	15	25	35	15	25	35	15
21	A	10	40	5	40	10	5	40	10	5	40	10	5
	B	30	15	5	25	15	10	25	15	10	25	15	10
22	A	5	10	40	5	10	40	10	40	5	10	40	5
	B	5	40	10	5	40	10	30	15	5	30	15	5
23	A	40	15	40	40	15	40	40	15	40	5	10	40
	B	5	35	25	5	35	25	5	35	25	5	40	10

Our design is comprised of choice menus with two treatment alternatives, and each treatment alternative is characterized by three attributes. Assuming that the location of alternative A and B does not affect the decision, it is the differences of attribute levels between the alternatives that is essential for the decision, that is, the difference of profit, Δ*π*, of health benefit, Δ*B*, and of patient consumption opportunities, Δ*C*. Each difference can be negative, zero, or positive, which leads to 3 × 3 × 3 incentive categories. Table E2 presents a breakdown of all 27 possible incentive categories.

**TABLE E2:** 27 possible incentive categories

Δ*C*	Δ*B*	Δ*π*		
		−	0	+
−	−	DOM	DOM	IV
−	0	DOM	DOM	II
−	+	V	III	VI
0	−	DOM	DOM	I
0	0	DOM	INDIFF	DOM
0	+	I	DOM	DOM
+	−	VI	III	V
+	0	II	DOM	DOM
+	+	IV	DOM	DOM

When all three differences are zero, the two treatment alternatives are identical (denoted as INDIFF in Table E2). When there is no trade‐off between any pair of attributes (i.e., all of the differences take the same sign whenever they are different from zero), one treatment alternative is dominant (denoted as DOM in Table E2). Choice menus without indifference and dominance can be grouped into six incentive structures with real world relevance:

- Trade‐off between *π* and *B*, and fixed *C*.
  Example: The physician can improve health by sacrificing his own profit. The patient is fully insured, or out‐of‐pocket payment is fixed across treatment alternatives.
- Trade‐off between *π* and *C*, and fixed *B*.
  Example: A choice between a brand‐named drug and a generic substitute, where the brand‐named drug is more costly for the patient and generates more profit for the provider.
- Trade‐off between *B* and *C*, and fixed *π*.
  Example: A salaried physician treats a patient without full insurance. Treatments with larger health effects result in higher out‐of‐pocket payments for the patient.
- Aligned incentive for *B* and *C*, and trade‐off between *π* and (*B*, *C*).
  Example: The physician can improve health by sacrificing his own profit (e.g., under a prospective payment). The patient's insurance covers treatments with larger health effects with lower out‐of‐pocket payment.
- Aligned incentive for *π* and *C*, and trade‐off between *B* and (*π*, *C*).
  Example: The physician can improve health by sacrificing his own profit (e.g., under a prospective payment). Treatments with larger health effects results in higher out‐of‐pocket payments for the patient.
- Aligned incentive for *π* and *B*, and trade‐off between *C* and (*π*, *B*).
  Example: The physician profits from health improvements (pay‐for‐performance). Treatments with larger health effects results in higher out‐of‐pocket payments for the patient.

One choice menu with a dominant alternative was deliberately chosen to be included in all blocks in the experiment; it is choice menu 15 in block 1, choice menu 20 in blocks 2, 3, and 4. Treatment B is strictly dominated because all three attributes take lower value than in Treatment A. The purpose of including a dominant choice menu is to check whether participants understand the experiment and act “rationally” (Johnson et al., 2019; Ryan et al., 2009). We found only 1 individual choosing the dominated alternative. All six incentive categories are represented in the experiment. Table E3 presents the corresponding incentive category of each choice menu in the experiment. The number of choice menus in each category is 11, 8, 9, 18, 19, and 11, respectively. Compared to previous designs which assume fully insured patients and have only included incentive category I (such as, Brosig‐Koch et al., 2017a; Hennig‐Schmidt et al., 2011), ours represents a much richer set of real‐world scenarios.

**TABLE E3:** Six incentive categories used in the experiment

Choice Menu	Block 1	Block 2	Block 3	Block 4
1	V	V	IV	V
2	II	I	IV	IV
3	VI	V	I	V
4	IV	I	I	III
5	III	II	IV	II
6	III	V	VI	V
7	III	VI	IV	III
8	IV	I	II	IV
9	IV	V	V	VI
10	III	IV	V	IV
11	VI	II	I	VI
12	VI	I	V	V
13	IV	IV	VI	II
14	VI	IV	II	I
15	DOM	VI	IV	V
16	I	V	V	III
17	IV	III	IV	V
18	IV	V	III	VI
19	V	V	II	I
20	I	DOM	DOM	DOM
21	I	VI	VI	VI
22	III	III	I	I
23	V	V	V	III

### E.4 Invitation letter (English)

To medical students at Shandong University:

**Invitation to participate in a decision experiment**

You are invited to participate in a health economic experiment. This experiment is part of a research collaboration between the Universities of Shandong (China), Oslo (Norway), and Cologne (Germany). The research is funded by the Research Council of Norway. With your participation, you support our research. You can earn money during the experiment, in addition to receiving 25 Yuan in participation fee.

The experiment consists of making decisions using pen and paper, and no prior knowledge is necessary. All information collected during the experiment is strictly anonymous and confidential and will only be used for the purpose of this research. We will not store any of your personal information. The experiment takes about 1.5 h, and will be carried out at 7:00 p.m., Tuesday April 4, in the Lecture Hall of School of Medicine. The participation is voluntary and you can withdraw from the experiment at any time.

Please contact Professor Wang for registration. Remember to bring your student ID to participate in the experiment.

Your sincerely,

Experiment organizers

Jian Wang,

School of Public Health, Shandong University

wangjiannan@sdu.edu.cn

Geir Godager,

Department of Health Management and Health Economics, University of Oslo

geir.godager@medisin.uio.no

Ge Ge,

Department of Health Management and Health Economics, University of Oslo

gege@medisin.uio.no

Daniel Wiesen,

Department of Business Administration and Health Care Management, University of Cologne

wiesen@wiso.uni‐koeln.de
